# Remarks on Pre-*ℐ*-Regular Pre-*ℐ*-Open Sets

**DOI:** 10.1155/2014/295198

**Published:** 2014-10-13

**Authors:** R. Sajuntha

**Affiliations:** Department of Mathematics, Ponjesly College of Engineering and Technology, Nagercoil, Tamil Nadu 629 003, India

## Abstract

We deal with the new class of pre-*ℐ*-regular pre-*ℐ*-open sets in which the notion of pre-*ℐ*-open set is involved. We characterize these sets and study some of their fundamental properties. We also present other notions called extremally pre-*ℐ*-disconnectedness, locally pre-*ℐ*-indiscreetness, and pre-*ℐ*-regular sets by utilizing the notion of pre-*ℐ*-open and pre-*ℐ*-closed sets by which we obtain some equivalence relation for pre-*ℐ*-regular pre-*ℐ*-open sets.

## 1. Introduction

The subject of ideals in topological space has been studied by Kuratowski [1] and Vaidyanathaswamy [2]. An ideal *ℐ* on a topological space (*X*, *τ*) is a nonempty collection of subsets of *X* which satisfies (i) *A* ∈ *ℐ* and *B* ⊂ *A* implies *B* ∈ *ℐ* and (ii) *A* ∈ *ℐ* and *B* ∈ *ℐ* implies *A* ∪ *B* ∈ *ℐ*. Given a topological space (*X*, *τ*) with an ideal *ℐ* on *X* and if *℘*(*X*) is the set of all subsets of *X*, a set operator (·)^⋆^ : *℘*(*X*) → *℘*(*X*), called a local function [1] of *A* with respect to *τ* and *ℐ*, is defined as follows: for *A* ⊂ *X*, *A* ^⋆^(*τ*, *ℐ*) = {*x* ∈ *X*∣*U*∩*𝒜* ∉ *ℐ*}, for every {*U* ∈ *τ*(*x*)} where *τ*(*x*) = {*U* ∈ *τ*∣*x* ∈ *U*}. A Kuratowski closure operator *cl* ^⋆^() for a topology *τ* ^⋆^(*X*, *τ*), called the ⋆-topology, finer than *τ* is defined by *cl* ^⋆^(*A*) = *A* ∪ *A* ^⋆^(*ℐ*, *τ*) [3]; when there is no chance for confusion, we will simply write *A* ^⋆^ for *A* ^⋆^(*ℐ*, *τ*) and *τ* ^⋆^ for *τ* ^⋆^(*ℐ*, *τ*). If *ℐ* is an ideal on *X*, then (*X*, *τ*, *ℐ*) is called an ideal space. A subset *A* of an ideal space (*X*, *τ*, *ℐ*) is said to be *ℐ*-open [4] if *A* ⊂ int⁡(*A* ^⋆^). A subset *A* of an ideal space (*X*, *τ*, *ℐ*) is said to be pre-*ℐ*-open [5] if *A* ⊂ int⁡(*cl* ^⋆^(*A*)). The complement of pre-*ℐ*-open set is called pre-*ℐ*-closed. The family of all pre-*ℐ*-open sets in (*X*, *τ*, *ℐ*) is denoted by *PℐO*(*X*, *τ*) or simply *PℐO*(*X*). Clearly *τ* ⊂ *PℐO*(*X*). The largest pre-*ℐ*-open set contained in *A*, denoted by *pℐ*int⁡(*A*), is called the pre-*ℐ*-interior of *A*. The smallest pre-*ℐ*-closed set containing *A*, denoted by *pℐcl*(*A*), is called the pre-*ℐ*-closure of *A*. A subset *A* of an ideal space (*X*, *τ*, *ℐ*) is said to be *α*-*ℐ*-open [6] if *A* ⊂ int⁡(*cl* ^⋆^(int⁡(*𝒜*))). The family of all *α*-*ℐ*-open sets is a topology finer than *τ*. We will denote the *α*-*ℐ*-interior subset of *A* of *X* by *αℐ*int⁡(*A*). A subset *A* of an ideal space (*X*, *τ*, *ℐ*) is *τ* ^⋆^-*dense* [7] if *cl* ^⋆^(*A*) = *X*. A space (*X*, *τ*, *ℐ*) is *ℐ*-*submaximal* [8] if every *τ* ^⋆^-*dense* subset of *X* is open.

The following lemmas will be useful in the sequel.

**Lemma 1 .**

Let (*X*, *τ*, *ℐ*) be an ideal space and let *A* be a subset of *X*. Then *pℐ*int⁡(*A*) = *A*∩int⁡(*cl* ^⋆^(*A*)) [9].

## 2. Pre-*ℐ*-Regular Pre-*ℐ*-Open Sets

A pre-*ℐ*-open set *A* of a space (*X*, *τ*, *ℐ*) is said to be* pre-ℐ-regular pre-ℐ-open* if *A* = *pℐ*int⁡(*pℐcl*(*A*)). The complement of a pre-*ℐ*-regular pre-*ℐ*-open set is called* pre-ℐ-regular pre-ℐ-closed set*, equivalently *A* = *pℐcl*(*pℐ*int⁡(*A*)). A subset *A* of a space (*X*, *τ*, *ℐ*) with an ideal *ℐ* is said to be pre-*ℐ*-regular if it is pre-*ℐ*-open and pre-*ℐ*-closed. Clearly, *X* and *∅* are pre-*ℐ*-regular pre-*ℐ*-open.

**Remark 2 .**

Also every pre-*ℐ*-regular set is pre-*ℐ*-regular pre-*ℐ*-open.

**Proof**

Assume *A* is pre-*ℐ*-regular that implies *A* is pre-*ℐ*-open and pre-*ℐ*-closed. *A* is pre-*ℐ*-open which implies *A* = *pℐ*int⁡(*A*) = *pℐ*int⁡(*pℐcl*(*A*)). Hence *A* is pre-*ℐ*-regular pre-*ℐ*-open.

But the converse is not true as shown by Example 3.

**Example 3 .**

Consider the ideal space (*X*, *τ*, *ℐ*) where *X* = {*a*, *b*, *c*}, *τ* = {*∅*, {*a*}, {*b*}, {*a*, *b*}, *X*}, and *ℐ* = {*∅*, {*a*}}. Here {*a*} is pre-*ℐ*-regular pre-*ℐ*-open but not pre-*ℐ*-closed.

Moreover, the intersection of two pre-*ℐ*-regular pre-*ℐ*-open sets is not pre-*ℐ*-regular pre-*ℐ*-open in general as Example 4 shows.

**Example 4 .**

Consider the ideal space (*X*, *τ*, *ℐ*) where *X* = {*a*, *b*, *c*}, *τ* = {*∅*, {*b*, *c*}, *X*}, and *ℐ* = {*∅*, {*a*}}. Here *PℐO*(*X*) = {*∅*, {*b*}, {*c*}, {*a*, *b*}, {*a*, *c*}, {*b*, *c*}, *X*}. Thus, *A* = {*a*, *b*}, *B* = {*a*, *c*} are both pre-*ℐ*-regular pre-*ℐ*-open. But *A*∩*B* is not, since it is not even pre-*ℐ*-open.

The notions of pre-*ℐ*-regular pre-*ℐ*-open and *ℐ*-open sets are independent of each other. Consider the space (*X*, *τ*) with an ideal *ℐ* as in Example 4. Here {*b*, *c*} is *ℐ*-open but not pre-*ℐ*-regular pre-*ℐ*-open. Consider the space *X* = {*a*, *b*, *c*} and *τ* = {*∅*, {*a*}, {*b*}, {*a*, *b*}, *X*}, *ℐ* = {*∅*, {*a*}}. Here {*a*} is pre-*ℐ*-regular pre-*ℐ*-open but not *ℐ*-open. Observe that every pre-*ℐ*-regular pre-*ℐ*-open set is pre-*ℐ*-open but the converse need not be true. Here {*b*, *c*} is pre-*ℐ*-open but not pre-*ℐ*-regular pre-*ℐ*-open.

**Theorem 5 .**

Let (*X*, *τ*, *ℐ*) be an ideal space and let *A*, *B* be any subsets of *X*. Then the following hold.

(a) If *A* ⊂ *B*, then *pℐ*int⁡(*pℐcl*(*A*)) ⊂ *pℐ*int⁡(*pℐcl*(*B*)).
(b) If *A* ∈ *PIO*(*X*), then *A* ⊂ *pℐ*int⁡(*pℐcl*(*A*)).
(c) For every *A* ∈ *PℐO*(*X*), *pℐ*int⁡(*pℐcl*(*pℐ*int⁡(*pℐcl*(*A*)))) = *pℐ*int⁡(*pℐcl*(*A*)).
(d) If *A* and *B* are disjoint pre-*ℐ*-open sets, then *pℐ*int⁡(*pℐcl*(*A*)) and *pℐ*int⁡(*pℐcl*(*B*)) are disjoint.
(e) If *A* is a pre-*ℐ*-regular pre-*ℐ*-open, then *pℐcl*(*X* − *A*) is pre-*ℐ*-regular pre-*ℐ*-closed.
(f) If *A* is pre-*ℐ*-regular pre-*ℐ*-open, then *pℐ*int⁡(*A*) is pre-*ℐ*-regular pre-*ℐ*-open.

**Proof**

(a) Suppose *A* ⊂ *B*⇒*pℐcl*(*A*) ⊂ *pℐcl*(*B*). Therefore *pℐ*int⁡(*pℐcl*(*A*)) ⊂ *pℐ*int⁡(*pℐcl*(*B*)).
(b) Suppose that *A* ∈ *PℐO*(*X*, *τ*)⇒*A* = *pℐ*int⁡(*A*) ⊂ *pℐ*int⁡(*pℐcl*(*A*)).
(c) It is obvious that *pℐ*int⁡(*pℐcl*(*A*)) ∈ *PℐO*(*X*, *τ*), so by (b) we have *pℐ*int⁡(*pℐcl*(*A*))⊂*pℐ*int⁡(*pℐcl*(*pℐ*int⁡(*pℐcl*(*A*)))). On the other hand *pℐ*int⁡(*pℐcl*(*A*)) ⊂ *pℐcl*(*A*) which implies *pℐcl*(*pℐ*int⁡(*pℐcl*(*A*)))⊂*pℐcl*(*pℐcl*(*A*)) = *pℐcl*(*A*). Therefore, *pℐ*int⁡(*pℐcl*(*pℐ*int⁡(*pℐcl*(*A*))))⊂*pℐ*int⁡(*pℐcl*(*A*)). Hence *pℐ*int⁡(*pℐcl*(*pℐ*int⁡(*pℐcl*(*A*)))) = *pℐ*int⁡(*pℐcl*(*A*)).
(d) Since *A* and *B* are disjoint pre-*ℐ*-open sets, we have *A*∩*B* = *∅* which implies *A*∩*pℐcl*(*B*) = *∅*⇒*A*∩*pℐ*int⁡(*pℐcl*(*B*)) = *∅*. Since *pℐ*int⁡(*pℐcl*(*B*)) is pre-*ℐ*-open, *pℐcl*(*A*)∩*pℐ*int⁡(*pℐcl*(*B*)) = *∅*. Hence*pℐ*int⁡(*pℐcl*(*A*))∩*pℐ*int⁡(*pℐcl*(*B*)) = *∅*.
(e) Given that *A* is pre-*ℐ*-regular pre-*ℐ*-open, *A* = *pℐ*int⁡(*pℐcl*(*A*)) implies *X* − *A* = *X* − *pℐ*int⁡(*pℐcl*(*A*)) = *pℐcl*(*pℐ*int⁡(*X* − *A*)). Therefore, *pℐcl*(*X* − *A*) = *pℐcl*(*pℐ*int⁡(*pℐcl*(*X* − *A*))). Hence *pℐcl*(*X* − *A*) is pre-*ℐ*-regular pre-*ℐ*-closed.
(f) By (e) if *A* is pre-*ℐ*-regular pre-*ℐ*-open, then *pℐcl*(*X* − *A*) is pre-*ℐ*-regular pre-*ℐ*-closed. Hence *X* − (*pℐcl*(*X* − *A*)) is pre-*ℐ*-regular pre-*ℐ*-open that implies *pℐ*int⁡(*A*) is pre-*ℐ*-regular pre-*ℐ*-open.

**Lemma 6 .**

For an ideal topological space (*X*, *τ*, *ℐ*) the following are equivalent.

(a) Every pre-*ℐ*-open set is open.
(b) Every *τ* ^⋆^-dense set is open.

**Proof**

(a) ⇒ (b): let *A* be a *τ* ^⋆^-dense set that implies *cl* ^⋆^(*A*) = *X* which implies int⁡(*cl* ^⋆^(*A*)) = *X*, so that *A* ⊂ int⁡(*cl* ^⋆^(*A*)) = *X*. By (a) every pre-*ℐ*-open set is open and hence *A* is open.

(b) ⇒ (a): let *B* be a pre-*ℐ*-open subset of *X*, so that *B* ⊂ int⁡(*cl* ^⋆^(*B*)) = *G*, say. Then *cl* ^⋆^(*B*) = *cl* ^⋆^(*G*), so that *cl* ^⋆^((*X* − *G*) ∪ *B*) = *cl* ^⋆^(*X* − *G*) ∪ *cl* ^⋆^(*B*) = (*X* − *G*) ∪ *cl* ^⋆^(*B*) = *X*, and thus (*X* − *G*) ∪ *B* is *τ* ^⋆^-dense in *X*. Thus (*X* − *G*) ∪ *B* is open. Now *B* = ((*X* − G) ∪ *B*)∩*G* is the intersection of two open sets, so that *B* is open.

**Lemma 7 .**

If a space (*X*, *τ*) with an ideal *ℐ* is *ℐ*-submaximal, then any finite intersection of pre-*ℐ*-open set is pre-*ℐ*-open.

**Proof**

From Lemma 6, every pre-*ℐ*-open set is open and hence a finite intersection of pre-*ℐ*-open set is pre-*ℐ*-open.

**Theorem 8 .**

If a space (*X*, *τ*) with an ideal *ℐ* is *ℐ*-submaximal, then any finite intersection of pre-*ℐ*-regular *p*-*ℐ*-open set is pre-*ℐ*-regular pre-*ℐ*-open.

**Proof**

Let {*O*_*i*_∣*i* = 1,2,…, *n*} be a finite family of pre-*ℐ*-regular pre-*ℐ*-open sets. Since the space *X* is *ℐ*-submaximal, then by Lemma 6, ∩{*O*_*i*_∣*i* = 1,2,…, *n*} is pre-*ℐ*-open. Therefore, ∩{*O*_*i*_∣*i* = 1,2,…, *n*}⊂*pℐ*int⁡(*pℐcl*(∩*O*_*i*_)). Also, for each *i* = 1,2,…, *n*, ∩*O*_*i*_ ⊂ *O*_*i*_ which implies *pℐ*int⁡(*pℐcl*(∩*O*_*i*_))⊂*pℐ*int⁡(*pℐcl*(*O*_*i*_)). Also, each *O*_*i*_ is pre-*ℐ*-regular pre-*ℐ*-open that implies *O*_*i*_ = *pℐ*int⁡(*pℐcl*(*O*_*i*_)) which implies *pℐ*int⁡(*pℐcl*(∩*O*_*i*_))⊂∩*O*_*i*_ and so ∩*O*_*i*_ = *pℐ*int⁡(*pℐcl*(∩*O*_*i*_)). Hence ∩*O*_*i*_ is pre-*ℐ*-regular pre-*ℐ*-open.

It should be noted that an arbitrary union of pre-*ℐ*-regular pre-*ℐ*-open set is pre-*ℐ*-regular pre-*ℐ*-open. But the intersection of two pre-*ℐ*-regular pre-*ℐ*-closed sets fails to be pre-*ℐ*-regular pre-*ℐ*-closed as shown by Example 9.

**Example 9 .**

Consider the ideal space (*X*, *τ*, *ℐ*) as in Example 3. Clearly, {*a*, *c*}, {*a*, *b*} are pre-*ℐ*-regular pre-*ℐ*-closed but their intersection is not pre-*ℐ*-regular pre-*ℐ*-closed.

**Theorem 10 .**

The following hold for a subset *A* of a space (*X*, *τ*, *ℐ*).

(a) If *A* is pre-*ℐ*-closed, then *pℐ*int⁡(*A*) is pre-*ℐ*-regular pre-*ℐ*-open.
(b) If *A* = *pℐ*int⁡(*A*), then *pℐcl*(*A*) is pre-*ℐ*-regular pre-*ℐ*-closed.
(c) If *A* and *B* are pre-*ℐ*-regular pre-*ℐ*-closed sets, then *A* ⊂ *B* if and only if *pℐ*int⁡(*A*) ⊂ *pℐ*int⁡(*B*).
(d) If *A* and *B* are pre-*ℐ*-regular pre-*ℐ*-open sets, then *A* ⊂ *B* if and only if *pℐcl*(*A*) ⊂ *pℐcl*(*B*).

**Proof**

(a) Since *A* is pre-*ℐ*-closed, *A* = *pℐcl*(*A*).

Now, *pℐ*int⁡(*pℐcl*(*pℐ*int⁡(*A*))) = *pℐ*int⁡(*pℐ*int⁡(*A*)) = *pℐ*int⁡(*A*). Hence *pℐ*int⁡(*A*) is pre-*ℐ*-regular pre-*ℐ*-open.

(b) Now *pℐcl*(*pℐ*int⁡(*pℐcl*(*A*))) = *pℐcl*(*pℐcl*(*A*)) = *pℐcl*(*A*). Hence *pℐcl*(*A*) is pre-*ℐ*-regular pre-*ℐ*-closed.

(c) Given that *A* and *B* are pre-*ℐ*-regular pre-*ℐ*-closed sets, therefore, *A* = *pℐcl*(*pℐ*int⁡(*A*)) and *B* = *pℐcl*(*pℐ*int⁡(*B*)). Clearly, *pℐ*int⁡(*A*) ⊂ *pℐ*int⁡(*B*) if *A* ⊂ *B*.

Conversely, *pℐ*int⁡(*A*) ⊂ *pℐ*int⁡(*B*). Now *A* = *pℐcl*(*pℐ*int⁡(*A*)) ⊂ *pℐcl*(*pℐ*int⁡(*B*)) ⊂ *B*. Hence *A* ⊂ *B*.

(d) Given that *A* and *B* are pre-*ℐ*-regular pre-*ℐ*-open, therefore, *A* = *pℐ*int⁡(*pℐcl*(*A*)) and *B* = *pℐ*int⁡(*pℐcl*(*B*)). Suppose *A* ⊂ *B*, *pℐcl*(*A*) = *pℐcl*(*pℐ*int⁡(*pℐcl*(*A*)))⊂*pℐcl*(*pℐ*int⁡(*pℐcl*(*B*)))⊂*pℐcl*(*B*). Therefore, *pℐcl*(*A*) ⊂ *pℐcl*(*B*).

Conversely, *pℐcl*(*A*) ⊂ *pℐcl*(*B*). Now *A* = *pℐ*int⁡(*pℐcl*(*A*)) ⊂ *pℐ*int⁡(*pℐcl*(*B*)) ⊂ *B*.

A subset *A* of an ideal topological space (*X*, *τ*, *ℐ*) is said to be *ℐ*-rare if it has no interior points in *τ* ^⋆^.

**Theorem 11 .**

Let (*X*, *τ*, *ℐ*) be an ideal space. Then the following hold.

(a) The empty set is the only subset which is nowhere dense and pre-*ℐ*-regular pre-*ℐ*-open.
(b) If *A* is pre-*ℐ*-regular pre-*ℐ*-closed, then every *ℐ*-rare set is pre-*ℐ*-open.

**Proof**

(a) Suppose *A* is nowhere dense and *A* is pre-*ℐ*-regular pre-*ℐ*-open. Then *A* = *pℐ*int⁡(*pℐcl*(*A*)) = *pℐcl*(*A*)∩int⁡(*cl* ^⋆^(*pℐcl*(*A*))), by Lemma 1. Therefore, *A* ⊂ *pℐcl*(*A*)∩int⁡(*cl* ^⋆^(*cl*(*A*)))⊂*pℐcl*(*A*)∩int⁡(*cl*(*A*)) = *pℐcl*(*A*)∩*∅* = *∅*.

(b) Suppose *A* is pre-*ℐ*-regular pre-*ℐ*-closed. Then *A* = *pℐcl*(*pℐ*int⁡(*A*)) = *pℐ*int⁡(*A*)∪*cl*(int⁡^⋆^(*pℐ*int⁡(*A*)))⊂*pℐ*int⁡(*A*) ∪ *cl*(int^⋆^(*A*)) = *pℐ*int⁡(*A*) ∪ *∅* = *pℐ*int⁡(*A*). Therefore, *A* = *pℐ*int⁡(*A*). Hence *A* is pre-*ℐ*-open.

An ideal space (*X*, *τ*, *ℐ*) is called extremally pre-*ℐ*-disconnected if the pre-*ℐ*-closure of every pre-*ℐ*-open set is pre-*ℐ*-open.

**Theorem 12 .**

For a topological space (*X*, *τ*, *ℐ*) the following are equivalent.

(a) (*X*, *τ*, *ℐ*) is extremally pre-*ℐ*-disconnected.
(b) Every pre-*ℐ*-regular pre-*ℐ*-open subset is pre-*ℐ*-regular.

**Proof**

(a) ⇒ (b): assume (*X*, *τ*, *ℐ*) is extremally pre-*ℐ*-disconnected. Suppose *A* is pre-*ℐ*-regular pre-*ℐ*-open. Then *A* is pre-*ℐ*-open and so *pℐcl*(*A*) is a pre-*ℐ*-open set. Hence *A* = *pℐ*int⁡(*pℐcl*(*A*)) = *pℐcl*(*A*). Hence *A* is pre-*ℐ*-closed which implies *A* is pre-*ℐ*-regular.

(b) ⇒ (a): suppose *A* is pre-*ℐ*-open. Then *pℐcl*(*A*) is pre-*ℐ*-regular pre-*ℐ*-closed which implies *X* − *pℐcl*(*A*) is pre-*ℐ*-regular pre-*ℐ*-open. Hence *X* − *pℐcl*(*A*) is pre-*ℐ*-regular. Therefore, *X* − *pℐcl*(*A*) is pre-*ℐ*-closed and so *pℐcl*(*A*) is pre-*ℐ*-open. Hence (*X*, *τ*, *ℐ*) is extremally pre-*ℐ*-disconnected.

**Theorem 13 .**

Let (*X*, *τ*, *ℐ*) be an extremally pre-*ℐ*-disconnected space and *A* ⊂ *X*. Then the following are equivalent:

(a) *A* is pre-*ℐ*-regular,
(b) *A* = *pℐcl*(*pℐ*int⁡(*A*)),
(c) *X* − *A* is pre-*ℐ*-regular pre-*ℐ*-open,
(d) *A* is pre-*ℐ*-regular pre-*ℐ*-open.

**Proof**

(a) ⇒ (b): suppose *A* is pre-*ℐ*-regular. Then *A* is pre-*ℐ*-open and pre-*ℐ*-closed and so *A* = *pℐ*int⁡(*A*) and *A* = *pℐcl*(*A*). Hence *A* = *pℐcl*(*pℐ*int⁡(*A*)).

(b) ⇒ (c): let *A* = *pℐcl*(*pℐ*int⁡(*A*)). Then *X* − *A* = *X* − *pℐcl*(*pℐ*int⁡(*A*)) = *pℐ*int⁡(*pℐcl*(*A*)) so *X* − *A* is pre-*ℐ*-regular pre-*ℐ*-open.

(c) ⇒ (d) is clear.

(d) ⇒ (a) follows from Theorem 12.

An ideal space (*X*, *τ*, *ℐ*) is called* locally pre-ℐ-indiscrete* if every pre-*ℐ*-open subset of *X* is pre-*ℐ*-closed (or) if every pre-*ℐ*-closed subset of *X* is pre-*ℐ*-open.

**Theorem 14 .**

Let (*X*, *τ*, *ℐ*) be an ideal space. Then the following are equivalent.

(a) (*X*, *τ*, *ℐ*) is locally pre-*ℐ*-indiscrete.
(b) Every pre-*ℐ*-open subset is pre-*ℐ*-regular.
(c) Every pre-*ℐ*-open subset is pre-*ℐ*-regular pre-*ℐ*-open.
(d) *pℐ*int⁡(*pℐcl*({*x*})) ≠ *∅*, for every *x* ∈ *X*.
(e) The empty set is the only nowhere dense subset of *X*.

**Proof**

(a) ⇒ (b): assume that (*X*, *τ*, *ℐ*) is locally pre-*ℐ*-indiscrete. Let *A* be a pre-*ℐ*-open subset of *X*. By hypothesis, *A* is pre-*ℐ*-closed. Hence *A* is pre-*ℐ*-regular.

(b) ⇒ (c): if *A* is pre-*ℐ*-open, then *A* = *pℐ*int⁡(*A*). Also by hypothesis, *A* is pre-*ℐ*-closed. Therefore, *A* = *pℐ*int⁡(*pℐcl*(*A*)). Hence *A* is pre-*ℐ*-regular pre-*ℐ*-open.

(c) ⇒ (d): since {*x*} is preopen, {*x*} is pre-*ℐ*-open. By (c), {*x*} is a pre-*ℐ*-regular pre-*ℐ*-open set. Therefore, {*x*} = *pℐ*int⁡(*pℐcl*({*x*})).

(d) ⇒ (e): by Theorem 11, in any space, the empty set is the only subset which is nowhere dense and pre-*ℐ*-regular pre-*ℐ*-open.

(e) ⇒ (a): suppose that *A* is a pre-*ℐ*-closed set. Now int⁡(*cl*(*A* − *pℐ*int⁡(*A*))) = int⁡(*cl*(*A* − (*A*∩int⁡(*cl* ^⋆^(*A*))))) = int⁡(*cl*(*A*∩(*X* − *A*)∪(int⁡(*cl* ^⋆^(*X* − *A*))))) = int⁡(*cl*(*A*∩(*X* − *A*)∪(*A* − int⁡(*cl* ^⋆^(*A*))))) = int⁡(*cl*(*A* − int⁡(*cl* ^⋆^(*A*))))⊂int⁡(*cl*(*A*) − int⁡(*cl* ^⋆^(*A*))) = int⁡(*cl*(*A*) − *cl*(int⁡(*cl* ^⋆^(*A*))))⊂int⁡(*cl*(*A*) − (int⁡(*cl* ^⋆^(*A*)))) = *∅*. Therefore *A* − *pℐ*int⁡(*A*) is nowhere dense which implies *A* = *pℐ*int⁡(*A*), and so *A* is pre-*ℐ*-open. Hence is *A* locally pre-*ℐ*-indiscrete.

An ideal space (*X*, *τ*, *ℐ*) is said to be *pℐR*-door if every subset of *X* is either pre-*ℐ*-regular pre-*ℐ*-open or pre-*ℐ*-regular pre-*ℐ*-closed.

**Theorem 15 .**

Let (*X*, *τ*, *ℐ*) be a *pℐR*-door space; then every pre-*ℐ*-open set in the space is pre-*ℐ*-regular pre-*ℐ*-open.

**Proof**

Let *A* be a pre-*ℐ*-open subset of *X*. Since *X* is *pℐR*-door, *A* is pre-*ℐ*-regular pre-*ℐ*-closed and so *A* = *pℐ*int⁡(*pℐcl*(*A*)) which implies that *pℐ*int⁡*A* = *pℐ*int⁡(*pℐ*int⁡(*pℐcl*(*A*))). Since *A* is pre-*ℐ*-open, *A* = p*ℐ*int⁡(*pℐcl*(*A*)). Hence *A* is pre-*ℐ*-regular pre-*ℐ*-open.

**Theorem 16 .**

Let (*X*, *τ*, *ℐ*) be an ideal space. A subset *A* of *X* is both *α*-*ℐ*-open and *α*-*ℐ*-closed; then *A* is a pre-*ℐ*-regular pre-*ℐ*-open set.

**Proof**

Let *A* be an *α*-*ℐ*-open and *α*-*ℐ*-closed set. Then *A* is a pre-*ℐ*-open and pre-*ℐ*-closed set and hence *A* is a pre-*ℐ*-regular pre-*ℐ*-open set.

**Theorem 17 .**

Let (*X*, *τ*, *ℐ*) be an ideal space. A subset *A* of *X* is *α*-*ℐ*-open and pre-*ℐ*-regular pre-*ℐ*-open; then *A* = int⁡(*cl* ^⋆^(int⁡(*A*))).

**Proof**

Suppose *A* is *α*-*ℐ*-open. Then *A* ⊂ int⁡(*cl* ^⋆^(int⁡(*A*))). And *A* is pre-*ℐ*-regular pre-*ℐ*-open which implies *A* = *pℐ*int⁡(*pℐcl*(*A*))⊃*pℐ*int⁡(*cl* ^⋆^(int⁡(*pℐcl*(*A*))))⊃*pℐ*int⁡(*cl* ^⋆^(int⁡(*A*)))⊃int⁡(*cl* ^⋆^(int⁡(*A*))). Therefore *A* = int⁡(*cl* ^⋆^(*A*)).
